# Nicotinate-Curcumin Impedes Foam Cell Formation from THP-1 Cells through Restoring Autophagy Flux

**DOI:** 10.1371/journal.pone.0154820

**Published:** 2016-04-29

**Authors:** Hong-Feng Gu, Hai-Zhe Li, Ya-Ling Tang, Xiao-Qing Tang, Xi-Long Zheng, Duan-Fang Liao

**Affiliations:** 1 Department of Physiology, University of South China, Hengyang, People's Republic of China; 2 Institute of Neuroscience, University of South China, Hengyang, People's Republic of China; 3 Department of Neurology of the First Affiliated Hospital, University of South China, Hengyang, People's Republic of China; 4 Smooth Muscle Research Group, Department of Biochemistry & Molecular Biology, Libin Cardiovascular Institute of Alberta, Faculty of Medicine, University of Calgary, Calgary, Alberta, Canada; 5 Division of Stem Cell Regulation and Application, State Key Laboratory of Chinese Medicine Powder and Medicine Innovation in Hunan, Hunan University of Chinese Medicine, Changsha, People's Republic of China; University of Milan, ITALY

## Abstract

Our previous studies have indicated that a novel curcumin derivate nicotinate-curcumin (NC) has beneficial effects on the prevention of atherosclerosis, but the precise mechanisms are not fully understood. Given that autophagy regulates lipid metabolism, the present study was designed to investigate whether NC decreases foam cell formation through restoring autophagy flux in oxidized low-density lipoprotein (ox-LDL)-treated THP-1 cells. Our results showed that ox-LDL (100 μg/ml) was accumulated in THP-1 cells and impaired autophagy flux. Ox-LDL-induced impairment of autophagy was enhanced by treatment with the autophagy inhibitor chloroquine (CQ) and rescued by the autophagy inducer rapamycin. The aggregation of ox-LDL was increased by CQ, but decreased by rapamycin. In addition, colocalization of lipid droplets with LC3-II was remarkably reduced in ox-LDL group. In contrast, NC (10 μM) rescued the impaired autophagy flux by significantly increasing level of LC3-II, the number of autophagolysosomes, and the degradation of p62 in ox-LDL-treated THP-1 cells. Inhibition of the PI3K-Akt-mTOR signaling was required for NC-rescued autophagy flux. Notably, our results showed that NC remarkably promoted the colocalization of lipid droplets with autophagolysosomes, increased efflux of cholesterol, and reduced ox-LDL accumulation in THP-1 cells. However, treatment with 3-methyladenine (3-MA) or CQ reduced the protective effects of NC on lipid accumulation. Collectively, the findings suggest that NC decreases lipid accumulation in THP-1 cells through restoring autophagy flux, and further implicate that NC may be a potential therapeutic reagent to reverse atherosclerosis.

## Introduction

Accumulation of macrophage foam cells within the arterial wall contributes to the pathogenesis of atherosclerosis and advanced plaque rupture [1, 2]. Foam cell accumulation may result from macrophage uptake of excessive modified lipoproteins or impairment of intracellular cholesterol efflux. Growing evidence suggests that promoting cholesterol efflux from these cells is an effective means to inhibit the development of atherosclerosis [3–5]. The first step of cholesterol efflux to apolipoprotein A-I (apoA-I) or high-density lipoprotein (HDL) is the release of cholesterol from lipid droplets (LDs) [6, 7]. Therefore, understanding how cholesterol esters in LDs are hydrolyzed and mobilized for efflux will help treat atherosclerotic disease.

Macroautophagy (hereafter referred to as autophagy) has been shown to be a major degradation route for abnormal aggregated proteins and damaged cellular organelles [8, 9]. The autophagic process is composed of the formation of double-membrane autophagosomes (APs) that sequester cytoplasmic components, fusion with lysosomes, and the degradation of autophagic cargoes in autophagolysosome (ALs). The above dynamic process of autophagy is defined as autophagy flux [10, 11]. Recently, some evidence supports that autophagy contributes to the degradation of intracellular modified low-density lipoproteins (LDLs) in foam cells [12–14]. In these foam cells, LDs are engulfed into APs and then delivered to lysosomes for degradation, followed by hydrolysis of intracellular lipids into free cholesterol mainly for ATP-binding cassette transporter A1 (ABCA1)-dependent efflux. Impaired autophagy flux can promote, but activation of autophagy impedes, the intracellular aggregation of lipids and formation of foam cells [12, 15–17]. Therefore, restoring the impaired autophagy flux in foam cell may be a promising therapeutic strategy to reverse atherosclerosis.

Curcumin, a hydrophobic polyphenol isolated from turmeric, was previously shown to protect human umbilical vein endothelial cells from oxidative stress injury via inducing activation of autophagy [18]. More recently, a series of curcumin derivatives have been developed to enhance protective effects on cardiovascular system and overcome the limitations of poor aqueous solubility and relatively low bioavailability [19, 20]. Nicotinate-Curcumin (NC), a compound synthesized from nicotinate and curcumin, exhibits superior water solubility and stability in solution. Importantly, the compound has been found to regulate lipid metabolism and inhibit atherosclerosis in apolipoprotein E deficient (apoE^-/-^) mice [21, 22]. However, the precise mechanisms underlying these protective effects remain obscure.

Here we hypothesized that NC can restore the impaired autophagy flux in oxidized low-density lipoprotein (ox-LDL)-treated THP-1 (an established human acute monocytic leukemia cell line) cells, and such a restoration may facilitate ox-LDL degradation and cholesterol efflux. In this study, we first investigated the protective function of autophagy against foam cell formation in ox-LDL-induced THP-1 cells. Then we focused on the effects of NC on autophagy flux and lipid accumulation in ox-LDL-treated THP-1 cells. Finally, we explored the mechanism by which NC decreased lipid aggregation and rescued the impaired autophagy flux in THP-1 cells challenged with ox-LDL. Our results indicate that NC decreases cholesterol ester accumulation in ox-LDL-induced THP-1 cells by facilitating autophagy flux likely through inhibition of the PI3k-Akt-mTOR pathway.

## Materials and Methods

### Cell culture

THP-1 cell line was obtained from the Cell Bank of the Chinese Academy of Sciences (Shanghai, China, CAS No: KG224) [23]. Cells were cultured in RPMI 1640 media (Sigma) containing 10% fetal bovine serum (FBS), penicillin (100 U/ml) and streptomycin (100 μg/ml) in 10 cm^2^ dishes at 37°C and 5% CO_2_. THP-1 monocyte differentiation into macrophages was induced by treatment with 160 mg/ml phorbol-12-myristate-13-acetate (PMA) for 24 h. Subsequently, macrophages were transformed into foam cells by incubation with 100 μg/ml ox-LDL for 36 h.

### Preparation of ox-LDL and Dil-ox-LDL

The ox-LDL was prepared as described [24]. Briefly, human LDL (purchased from Dalian Meilun Biotech Co., CAS No: MB12475) was incubated with 5 μM CuSO_4_ for 24 h at 37°C. The extent of oxidation was analyzed by measuring thiobarbituric acid-reactive substances (TBARs). TBARS was determined colorimetrically with malondialdehyde (MDA) as a standard. The TBARS of starting LDL is 0.15 nM of MDA/mg protein, and ox-LDL is 28.6 nM of MDA/mg protein.

Dil-ox-LDL was prepared with purified ox-LDL as described previously [25].

### Oil Red O staining

Cultured THP-1 cells were plated at density of 2 × 10^5^ cells/well on cover slides in six-well plates and incubated with noted reagents for 36 h. Cells were fixed with 4% paraformaldehyde after washing 3 times with cold phosphate-buffered saline (PBS), and then stained with 0.5% Oil Red O solution. Hemotoxylin was used for counterstaining. Foam cells were observed under a microscope at × 200 magnification. Total intracellular cholesterol levels were measured as described [26].

### Cholesterol efflux assay

THP-1 cells were plated in 12-well plates at density of 1 × 10^6^ cells/well, and then incubated in medium containing 100 μg/ml ox-LDL that was labeled with 0.5 μ Ci/ml ^3^H-cholesterol (PerkinElmer) for an additional 30 h. Subsequently, macrophages were washed twice with PBS and incubated with 2 mg/ml BSA (FAFA, Sigma) media. Cholesterol efflux was measured in the presence of human apoA-I (10 mg/ml) or HDL (50 mg/ml) in serum-free media with or without the indicated treatments for 6 h. Supernatants were collected for cholesterol efflux assay. The results were expressed as a percentage of effluxed ^3^H-cholesterol / total cell cholesterol ^3^H-cholesterol content (effluxed ^3^H-cholesterol + intracellular ^3^H-cholesterol) × 100%.

### Cholesterol uptake assay

Dil-ox-LDL binding assay was used to measure cholesterol uptake by THP-1 macrophages. Cells were treated with Dil-ox-LDL (100 μg/ml), Dil-ox-LDL (100 μg/ml) + NC (10 μM), ox-LDL (100 μg/ml) + NC (10 μM) + 3-MA (10 mM), Dil-ox-LDL (100 μg/ml) + NC (10 μM) + CQ (20 μM), and Dil-ox-LDL (100 μg/ml) + vehicle for 36 h, respectively. After cells were washed 3 times with PBS, the cell lysates were harvested and analyzed by the fluorometry as described previously [27].

### Western blot analysis

Western blot analyses were performed as described [28]. In brief, cells were lysed with Laemmli buffer. Proteins (20 μg from each sample) were separated by SDS-polyacrylamide gels (Invitrogen), followed by electrophoretical transferring to polyvinylidenedifluoride (PVDF) membranes. After being blocked with 5% milk, the membranes were first incubated with primary antibodies against LC3, p62, p-mTOR, p-p70S6K, and PI3K, respectively, and then washed and incubated with horseradish peroxidase-linked secondary antibodies. The bands were visualized using an enhanced chemiluminescence kit. Densitometry of each band was analyzed with Sigma Scan Pro5 software and normalized to that of GAPDH.

### Transmission electron microscopy (TEM)

Ox-LDL-loaded THP-1 macrophages were incubated with indicated reagents for 36 h. After removal of culture medium and washing three times, cells were fixed with 2.5% glutaraldehyde, postfixed in 1% OsO_4_ and dehydrated in a graded ethanol series. Samples were embedded in epoxy resin, followed by ultra-thin sectioning (40–60 nm). The sections were counter-stained with uranyl acetate and lead citrate, and observed at 60 kV by a JEOL 1230 TEM with images acquired.

### Cell transfection

THP-1 cells were transfected with a GFP-LC3II or GFP-RFP-LC3II plasmid (Han-heng company, Shanghai, China) according to the manufacturer’s instructions. Briefly, cells were plated on coverslips in 24-well plates (2 × 10^5^ cells/well) overnight, and cells were then transfected with the purified recombinant plasmid GFP-LC3II or GFP-RFP-LC3II using Lipofectamine LTX and PLUS reagents (Invitrogen) for 4 h in Opti-MEM medium (Invitrogen). Subsequently, the transfection medium was replaced, and the cells were cultured in DMEM complete medium containing 15% (vol/vol) FBS for 24 h, followed by various treatments as indicated in figure legends.

### Confocal microscopy

The images of THP-1 cells transfected with GFP-RFP-LC3II plasmid were obtained with a Leica TCS SP5 laser scanning confocal microscope. To quantify the number of autophagic puncta (GFP/RFP, free RFP) per cell, confocal microscopy images were binarized to black and white images by Quantity one (Bio-Rad) and then converted to centroids for scoring automatically by the image processing tool kit.

### Immunocytochemistry

To evaluate intracellular lipid droplet accumulation, THP-1 cells or the cells transfected with a GFP-LC3II plasmid were grown on coverslips in six-well plates and incubated with indicated reagents for 36 h. Cells were washed with PBS 3 times and fixed with 4% paraformaldehyde/PBS. Cells were then stained with Nile Red (10 ng/ml) for 30 min to visualize LDs. Images were obtained using a confocal microscope with appropriate lasers. 15 cells were randomly selected from each group to measure the average number of LDs and the percentage of colocalization of LDs with LC3-II per cell.

### Statistical analysis

All data were expressed as mean ± SEM of at least 3 independent experiments. Differences between the groups were determined by one-way ANOVA with Newman-Keuls post-hoc test. Means between two groups were analyzed by Student’s t-test. Statistics was performed using the GraphPad Prism 5 software. *P* < 0.05 was considered to be statistical significance.

## Results

### Autophagy protects against foam cell formation in ox-LDL-treated THP-1 macrophages

**Fig 1:**
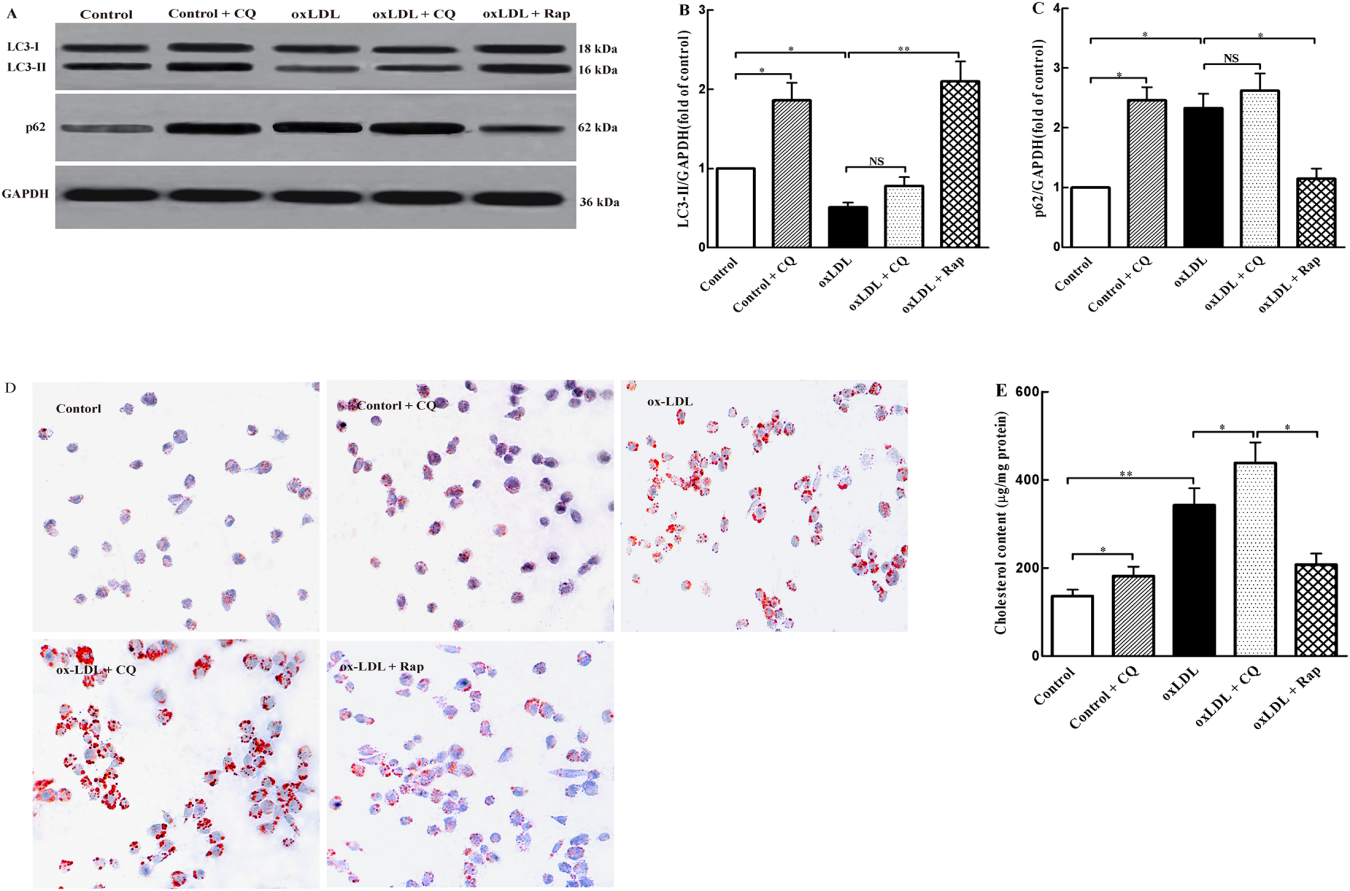
Autophagy prevents ox-LDL-induced foam cell formation in THP-1 cells. THP-1 cells were treated with the vehicle solution (control), control + CQ (10 μM), ox-LDL (100 μg/ml), ox-LDL (100 μg/ml) + CQ (10 μM), ox-LDL (100 μg/ml) + Rap (20 μM) for 36 h, respectively. (A) LC3-I (18 kDa), LC3-II (16 kDa), and p62 (62 kDa) protein levels were detected by western blot analysis. Each lane contained 20 μg proteins for all experiments. (B) and (C) The LC3-II/LC3-I ratio and p62 level were quantified with Sigma Scan Pro5 software. Each lane was normalized to that of GAPDH (kDa). (D) Oil red O staining was used to evaluate THP-1 foam cell formation (magnification × 200). (D) Intracellular total cholesterol content was determined by enzymatic assay. All the data were shown as mean ± SEM of three independent experiments. *P < 0.05, **P < 0.01.

To explore the role of autophagy in THP-1 foam cell formation, we evaluated the autophagic changes in ox-LDL-treated THP-1 cells. LC3-II and p62 proteins are two markers of autophagy. We first investigated whether the levels of these two proteins were altered in THP-1cells in response to treatment with ox-LDL. We observed that treatment with ox-LDL significantly decreased LC3-II expression (Fig 1A and 1B) and increased p62 levels (Fig 1A and 1C) in THP-1 cells. To confirm a block in autophagy flux, the fusion between autophagosomes and lysosomes was inhibited with Chloroquine (CQ, an inhibitor of autophagy flux). Our results showed that LC3-II and p62 levels were remarkably increased in control + CQ group as compared to the control group. However, ox-LDL group had nearly saturated LC3-II and p62 levels, and no further significant increase was observed in response to treatment with CQ, suggesting a defect in autophagy flux.

We then assessed the effects of autophagy on foam cell formation in ox-LDL-treated THP-1 cells. CQ and rapamycin (Rap, an inhibitor of mTOR) were used to block and induce autophagy in THP-1 cells, respectively. As shown in Fig 1A and 1C, CQ exacerbated, whereas Rap rescued, the impairment of autophagy in ox-LDL-treated THP-1 cells, as indicated by changes of LC3II and p62 levels. Consistently, both Oil Red O staining and total cholesterol quantification showed that CQ promoted the ox-LDL-induced foam cell formation and increased intracellular cholesterol content, whereas Rap exerted the opposite effects (Fig 1D and 1E). These results suggested that autophagy attenuated foam cell formation in ox-LDL-treated THP-1 cells.

### NC rescues the impaired autophagy flux in ox-LDL-treated THP-1 cells

**Fig 2:**
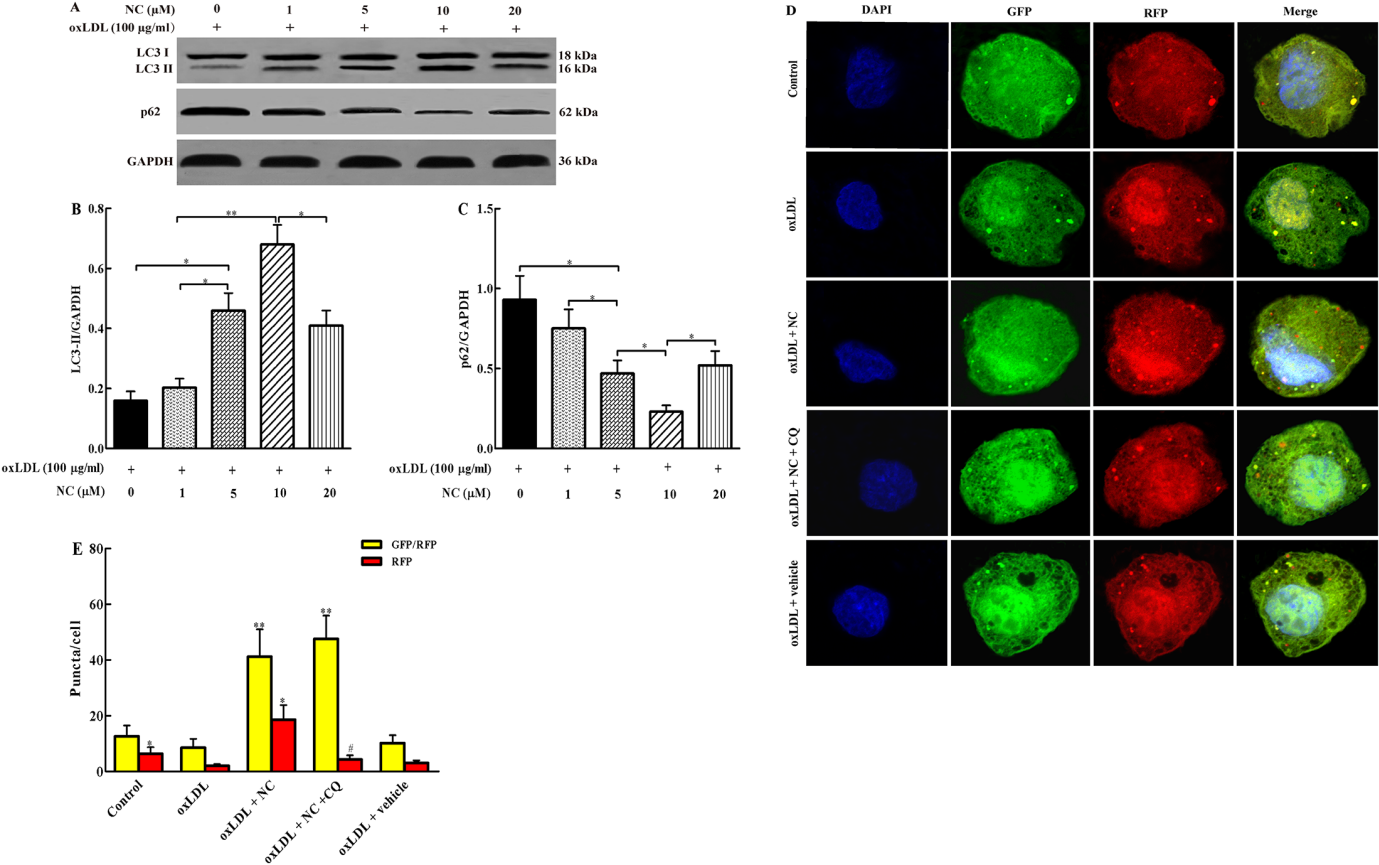
NC rescues the impaired autophagy flux in ox-LDL-treated THP-1 cells. (A) THP-1 cells were treated with different concentration of NC (0, 1, 5, 10, 20 μM) in the presence of ox-LDL (100 μg/ml) for 36 h. Cell lysates were analyzed by western blotting assay for LC3-I (18 kDa), LC3-II (16 kDa), and p62 (62 kDa) protein levels. Each lane was loaded with 20 μg proteins in all experiments. (B) and (C) The LC3-II and p62 levels were quantified with Sigma Scan Pro5 software. (D) Confocal images of representative images of GFP and RFP fluorescent puncta in THP-1 cells transfected with GFP-RFP-LC3II for 24 h, and then treated with indicated reagents for 36 h. (E) Quantification of GFP/RFP double-positive and RFP single-positive puncta in each cell treated with indicated reagents for 36 h (n = 23 cells/group). All the data were shown as mean ± SEM of three independent experiments. *P < 0.05, **P < 0.01.

Autophagy flux was suppressed in ox-LDL-treated THP-1 cells. Subsequently, we determined the effects of NC on the impaired autophagy. THP-1 cells were treated with ox-LDL (100 μg/ml) in the presence or absence of NC for 36 h, followed by determination of the levels of LC3II and p62 with western bolt assay. As shown in Fig 2A and 2C, the ox-LDL-induced autophagy flux impairment was significantly rescued by NC in a concentration-dependent manner (1, 5, 10 μmol/L) with the maximal effect at 10 μM, as demonstrated by the elevated LC3II and decreased p62 levels. Therefore, 10 μM NC was selected for cell treatments in subsequent experiments. Taken together, our data suggested that NC rescued the impaired autophagy flux in ox-LDL-treated THP-1 cells.

**Fig 3:**
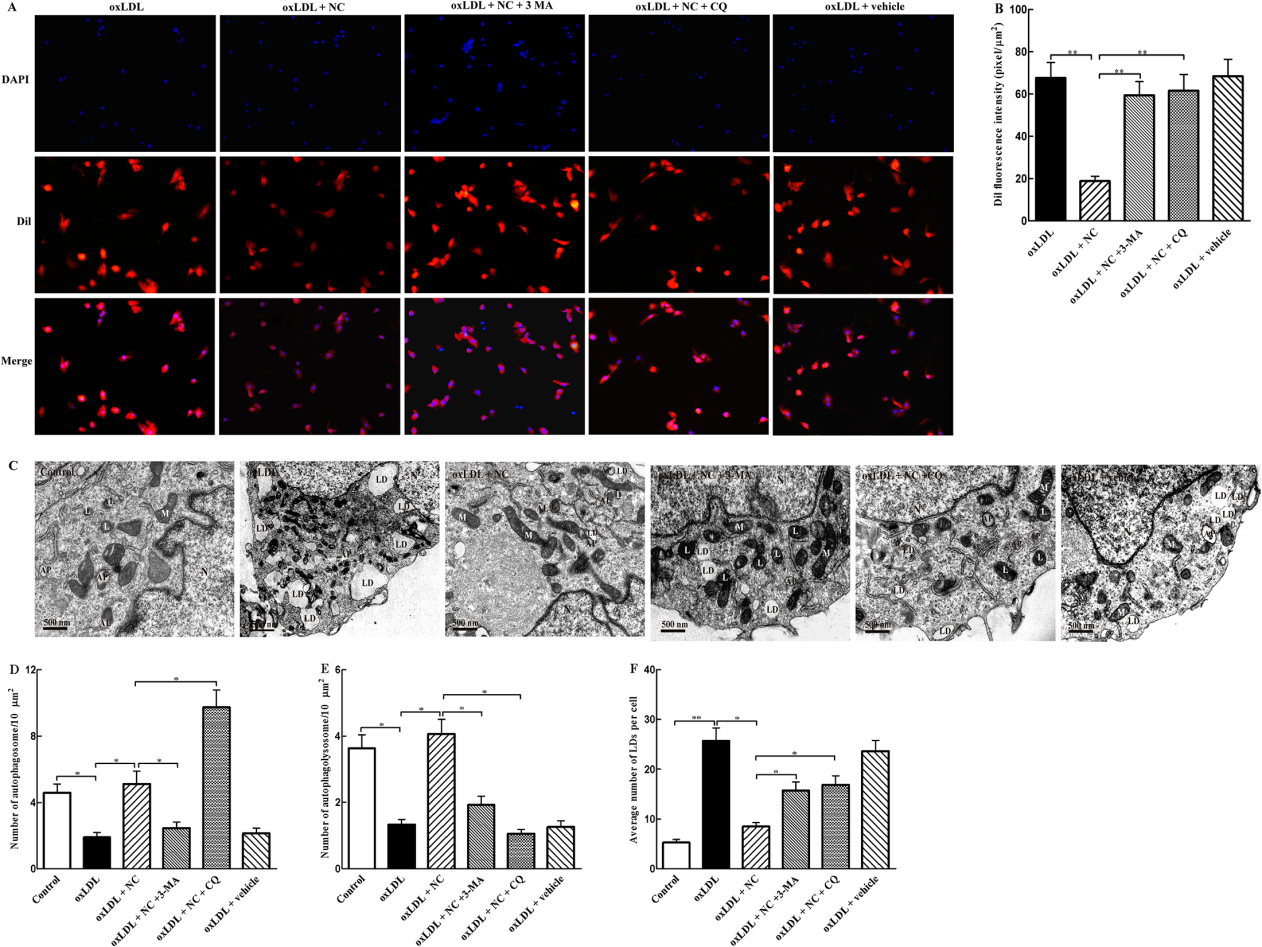
NC reduces ox-LDL accumulation in THP-1 cells via activation of autophagy. (A) Representative photomicrographs of THP-1 cells loaded with Dil-ox-LDL. Cells were treated with ox-LDL (100 μg/ml), ox-LDL (100 μg/ml) + NC (10 μM), ox-LDL (100 μg/ml) + NC (10 μM) + 3-MA (10 mM), ox-LDL (100 μg/ml) + NC (10 μM) + CQ (20 μM), and ox-LDL (100 μg/ml) + vehicle for 36 h. After washing 3 times, cell lysates were collected for the measurement of fluorescence. Nuclei were counterstained with DAPI. (B) Quantification of fluorescence intensity from experiments as described in (A). (C) TEM was used to evaluate foam cell formation and autophagy alteration. THP-1 cells were treated with vehicle, ox-LDL (100 μg/ml), ox-LDL + NC (10 μM)), ox-LDL (100 μg/ml) + NC (10 μM) + 3-MA, ox-LDL (100 μg/ml) + NC (10 μM) + CQ (20 μM), and ox-LDL (100 μg/ml) + vehicle for 36 h, respectively. Mitochondria (M), the nucleus (N), lysosomes (L), autophagosomes (APs), autophagolysosomes (ALs), and lipid droplets (LDs) were indicated. (D, E, and F) Average number of APs, ALs, and LDs was quantified as described in Methods section (n = 12 cells/group). All the data were shown as mean ± SEM of 3 independent experiments. *P < 0.05, **P < 0.01.

To further examine whether NC restored autophagy flux in ox-LDL-treated THP-1 cells, the cells were overexpressed with the tandem monomeric GFP-RFP-LC3-tagged protein and analyzed by confocal microscopy. The GFP fluorescence (green puncta) primarily indicates autophagosome, whereas RFP fluorescence (red puncta) represents both APs and ALs. Given that mCherry fluorescence persists even in acidic condition of the lysosome lumen where GFP loses its fluorescence, colocalization of GFP and RFP fluorescence (yellow puncta) in merged images indicated APs, whereas the solely red puncta represented ALs. Autophagy flux was indicated by the ratio between the number of red and yellow dots. As shown in Fig 3D and 3E, autophagy flux in ox-LDL group was much lower than that in control group, suggesting an impairment of autophagy flux in THP-1 cells by ox-LDL treatment. In ox-LDL + NC group, both green and red puncta were obviously increased, and the yellow dots were also remarkably increased in the merged images when compared with the ox-LDL group. In a comparison of ox-LDL + NC group, there were much more yellow puncta in ox-LDL + NC +CQ group and much less red puncta. These results indicated that treatment with NC restored the autophagy flux in ox-LDL-treated THP-1 cells.

### NC reduces ox-LDL accumulation in THP-1 cells through induction of autophagy

It was previously showed that autophagy deficiency promotes atherosclerosis, and curcumin induces autophagy and increases vascular endothelial cell survival in the presence of oxidative stress. Thus we speculated that NC treatment may decrease ox-LDL accumulation in THP-1 cells by restoring autophagy flux. To investigate whether NC inhibition of THP-1 foam cell formation was dependent on autophagy flux, we then assessed whether the inhibition of autophagy by 3-methyladenine (3-MA) or blockade of autophagy-lysosome pathway by CQ would affect the NC-induced protection in THP-1 cells. We took advantage of Dil-ox-LDL, an ox-LDL labeled with the fluorescent probe, which can be up-taken and aggregated by macrophages. As shown in Fig 3A and 3B, the accumulation of ox-LDL was much more significant in ox-LDL-treated group than ox-LDL + NC group, suggesting that treatment with 10 μM NC had significant effects (*P* < 0.01). Compared with ox-LDL + NC group, however, the intracellular accumulation of Dil-ox-LDL was more significant (*P* < 0.01) in ox-LDL + NC + 3-MA or ox-LDL + NC + CQ group, suggesting that co-treatment with 3-MA or CQ abrogated the inhibitory effect of NC on ox-LDL aggregation in THP-1 cells. These results suggest that NC diminishes ox-LDL accumulation in THP-1 cells likely through restoring autophagy flux.

To further establish that NC inhibits foam cell formation in ox-LDL-treated THP-1 cells via rescuing autophagy flux, we used the previously described morphological criteria to define AP, AL, and LD with transmission electron microscopy (TEM). As shown in Fig 3C, TEM images showed obvious morphologic changes in ox-LDL-treated THP-1 cells, when compared with the control cells, characterized by increased number of LDs, impaired organelles, and decreased number of APs and ALs. In contrast, NC treatment markedly decreased the accumulation of LDs, and increased the number of ALs and APs in ox-LDL-treated THP-1 cells. Meanwhile, double-membrane vesicles, which are analogous to APs, in and around LDs were observed in ox-LDL + NC group. However, compared with ox-LDL + NC group, the numbers of APs (Fig 3C and 3D) and ALs (Fig 3C and 3E) were significantly decreased in ox-LDL + NC + 3-MA group. The number of APs (Fig 3C and 3D) significantly increased, and the number of ALs (Fig 3C and 3E) reduced in ox-LDL + NC + CQ group when compared with those in ox-LDL + NC group, accompanied by significant enhancement of LD accumulation (Fig 3A and 3F). These data further suggested that NC treatment decreased LD aggregation in THP-1 cells via rescuing autophagy.

### NC facilitates lipophagy of THP-1 cells

**Fig 4:**
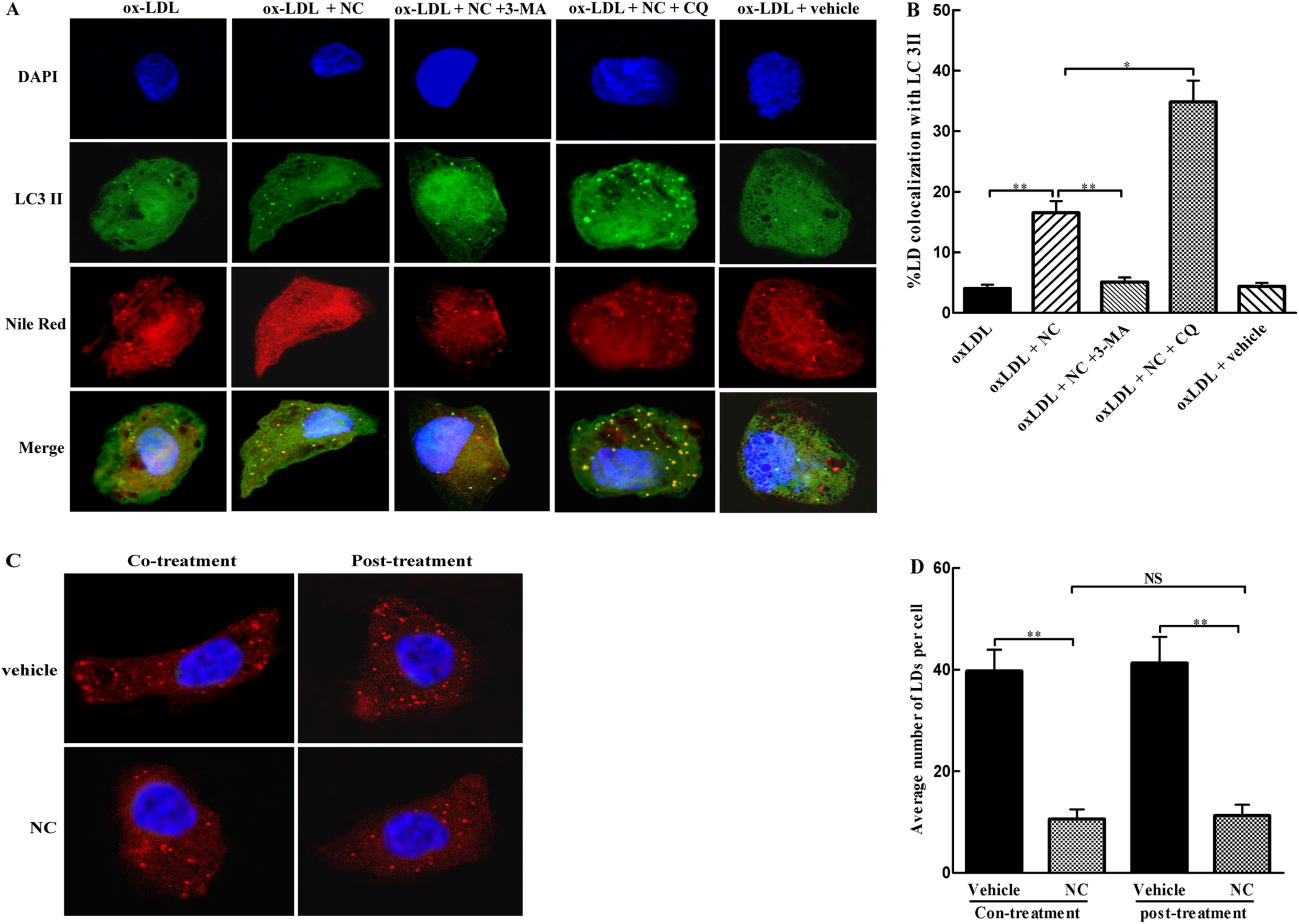
NC enhances intracellular ox-LDL degradation through facilitation of lipophagy. (A) Representative photomicrographs of colocalization of lipid droplets (LDs) with LC3-II in THP-1 cells. After THP-1 cells were transfected with GFP-LC3II for 24 h, cells were treated with ox-LDL (100 μg/ml), ox-LDL (100 μg/ml) + NC (10 μM), ox-LDL (100 μg/ml) + NC (10 μM) + 3-MA (10 mM), ox-LDL (100 μg/ml) + NC (10 μM) + CQ (20 μM), and ox-LDL (100 μg/ml) + vehicle for 36 h. After washing with PBS, cells were fixed with 4% paraformaldehyde, and then stained with Nile Red (10 ng/ml) for 30 min to evaluate the accumulation of LDs. The colocalization of LDs with LC3II was examined by immunocytochemistry as described in Methods section. (B) The percentage of colocalization of LDs with LC3-II was quantified with Image J software (n = 16 cells/group). (C) Representative photomicrographs of LD accumulation in THP-1cells. Cells were incubated with ox-LDL (100 μg/ml) conjugate without or with NC of 36 h, or pre-incubated with ox-LDL for 4 h, and then treated without or with NC for additional 36 h. The intracellular LD accumulation was evaluated by Nile Red (10 ng/ml) staining. (D) Average number of LDs in THP-1 cells was quantified (n = 12 cells/group). All the data were shown as mean ± SEM of 3 independent experiments. NS: no significant difference. *P < 0.05, **P < 0.01.

To examine whether degradation of LDs is associated with autophagy, the colocalization of LC3-II with ox-LDL was determined by immunofluorescence. As indicated in Fig 4A and 4B, there was only a small number of colocalization of ox-LDL with LC3-II in ox-LDL-treated THP-1cells, indicating that ox-LDL degradation by autophagy was inhibited. Interestingly, more colocalization of ox-LDL with LC3-II was found in ox-LDL + NC group than the control group, which was further significantly (*P* < 0.05) enhanced in ox-LDL + NC + CQ as compared with that in ox-LDL + NC group. Compared with ox-LDL + NC group, however, colocalization of these two markers was remarkably reduced in ox-LDL + NC + 3-MA group, suggesting that inhibition of autophagy decreased ox-LDL degradation. These data suggest that NC promotes the degradation of cytoplasmic ox-LDL via enhancing autophagy.

To further investigate whether NC decreased foam cell formation via facilitating lipophagy or inhibiting formation of lipid droplets, THP-1 cells were incubated with ox-LDL together with or without NC, or pre-incubated with ox-LDL, followed by treatment with or without NC. The number of LDs in THP-1 cells was counted. As shown in Fig 4C and 4D, the number of LDs in NC-treated group was significantly reduced when compared with that in vehicle group. However, the number of LDs in the group with NC co-treatment was not significantly different as compared with that in the group with NC treatment after ox-LDL incubation. Collectively, these results indicate that NC-induced decrease in LD accumulation was mainly via facilitation of lipophagy, but not inhibition of its formation.

### NC promotes cholesterol efflux from THP-1 cells via rescuing autophagy flux

**Fig 5:**
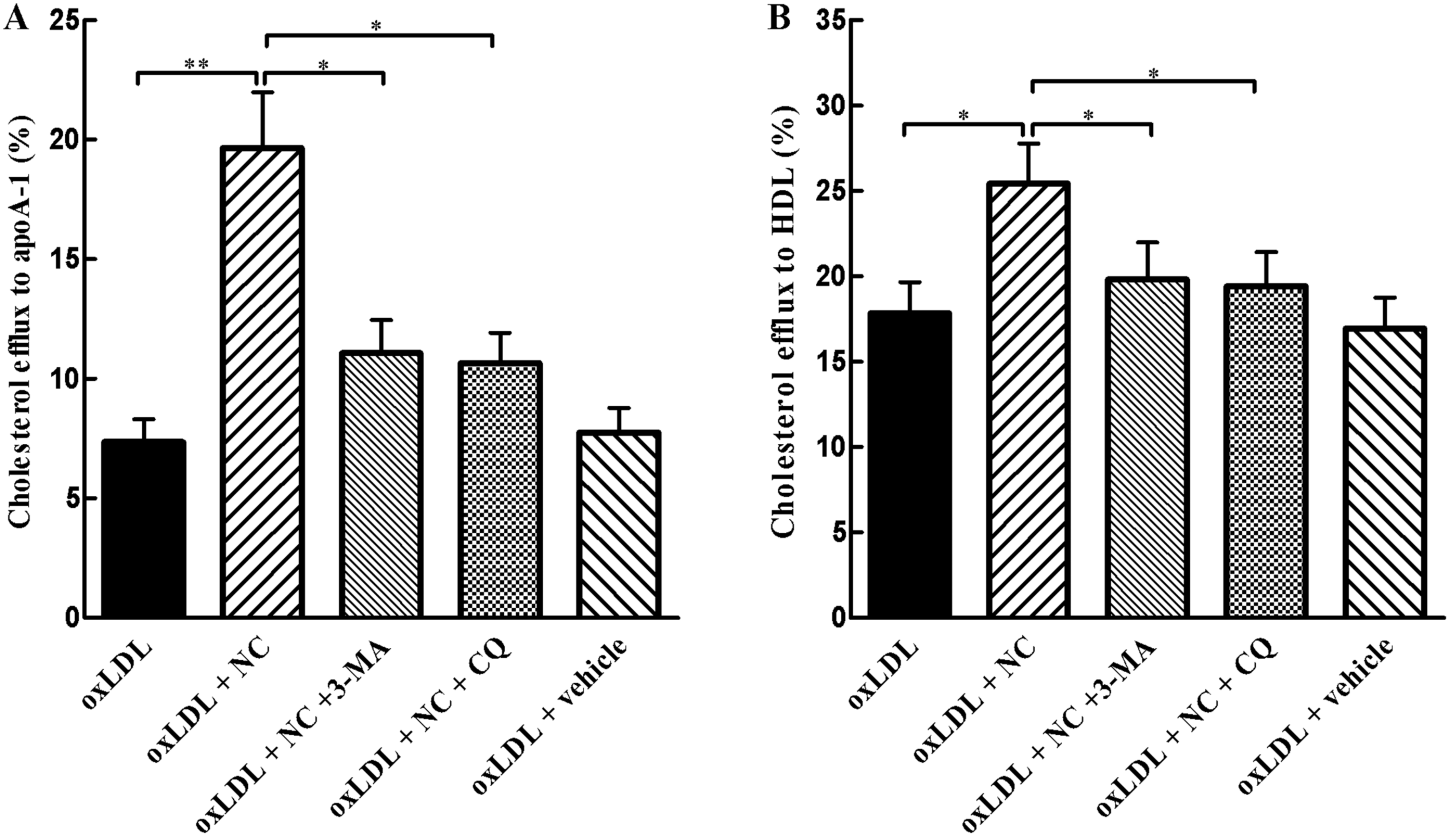
NC promotes cholesterol efflux via restoring autophagy flux. (A)and (B) THP-1 cells were incubated in medium containing 100 μg/ml ox-LDL that was labeled with 0.5 μ Ci/mL ^3^H-cholesterol (PerkinElmer) for an additional 30 h and then treated with vehicle, NC (10 μM), NC (10 μM) +3-MA (10 mM), and NC (10 μM) + CQ (20 μM) for additional 6 h. Subsequently, ApoA1- or HDL-mediated cholesterol efflux was analyzed by liquid scintillation counting assay. The efflux is expressed as the percentage of effluxed ^3^H-cholesterol/total cell cholesterol ^3^H-cholesterol content (effluxed ^3^H-cholesterol + intracellular ^3^H-cholesterol) × 100%. All the data were shown as mean ± SEM of 3 independent experiments. NS: no significant difference. *P < 0.05, **P < 0.01.

It has been established that autophagy regulates lipid metabolism and cholesterol efflux. Since NC restored autophagy flux and decreased foam cell formation in ox-LDL-treated THP-1 cells, we next explored whether NC promotes cholesterol efflux via rescuing autophagy. In present study, THP-1 cells were loaded with ox-LDL containing ^3^H-cholesterol, and the efflux of the labeled cholesterol to apoA-1 and HDL was determined, respectively. As shown in Fig 5A, apoA1-mediated cholesterol efflux was significantly (*P* < 0.01) increased in ox-LDL + NC group, as compared with THP-1 cells treated with ox-LDL alone. However, compared with ox-LDL + NC group, apoA1- and HDL-mediated cholesterol efflux was dramatically decreased in ox-LDL + NC + 3-MA group, respectively, implying that 3-MA diminished the effect of NC on cholesterol efflux in THP-1 cells. Furthermore, the effect of CQ on NC-promoted cholesterol efflux mediated by apoA1 and HDL was also examined. Our results showed that CQ had similar effects as shown in the group treated with ox-LDL + NC + 3-MA (Fig 5B). These findings suggest that NC increases cholesterol efflux via restoring autophagy flux in ox-LDL-treated THP-1 cells.

### NC restores autophagy flux through the m-TOR pathway

**Fig 6:**
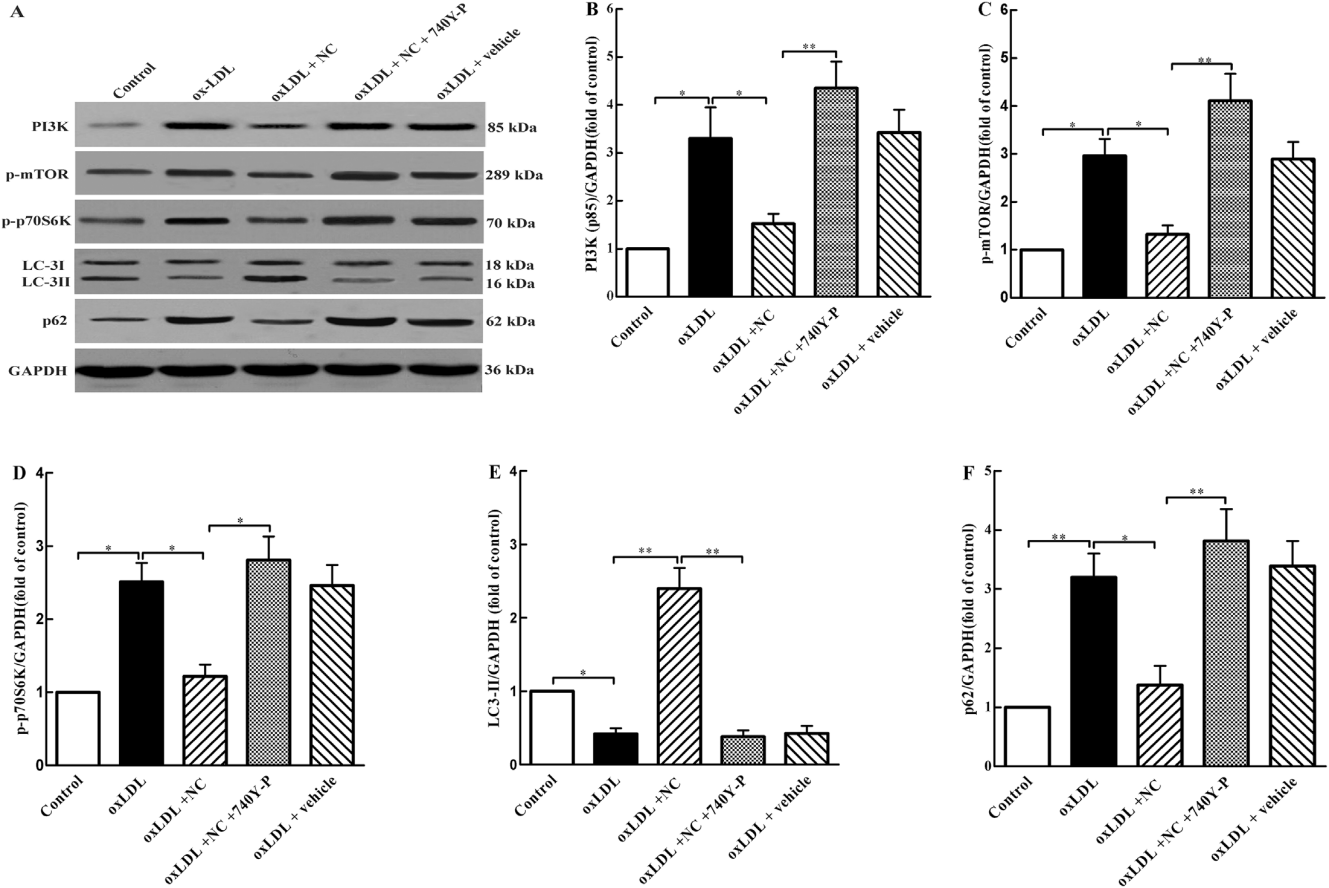
NC rescues autophagy flux via inhibiting the PI3K /m-TOR pathway. (A) THP-1 cells were treated with vehicle, ox-LDL (100 μg/ml), ox-LDL (100 μg/ml) +NC (10 μM), ox-LDL (100 μg/ml) +NC (10 μM) + 740Y-P (20 μM), and ox-LDL (100 μg/ml) + vehicle for 36 h. Cell lysates were collected and analyzed by western blotting assay for PI3K (85 kDa), p-mTOR (289 kDa), p-p70S6K (70 kDa), LC3-I (18 kDa), LC3-II (16 kDa), and p62 (62 kDa) protein levels. Each lane was loaded with 20 μg proteins for all experiments. (B, C, D, and E) The relative optical density values of PI3K, p-mTOR, p-p70S6K, LC3-II, LC3-I, and p62 to GAPDH, respectively, were quantified with Sigma Scan Pro5 software. All the data were shown as mean ± SEM of 3 independent experiments. NS: no significant difference. *P < 0.05, **P < 0.01.

It is known that the m-TOR signaling plays a critical role in regulation of autophagy and mTOR inhibition leads to activation of autophagy. To understand the potential mechanism underlying NC restoration of autophagy flux in ox-LDL-treated THP-1 cells, we further investigated the effect of NC on ox-LDL-induced mTOR activation and impairment of autophagy flux by western blot assay. As shown in Fig 6, the levels of PI3K (Fig 6A and 6B), phosphorylated mTOR (p-mTOR) (Fig 6A and 6C), p-p70S6K (Fig 6A and 6D), and p62 (Fig 6A and 6F) were significantly increased in ox-LDL group compared with control group, but the expression of LC3-II (Fig 6A and 6E) was markedly reduced in ox-LDL group, suggesting that treatment with ox-LDL activates the PI3K /m-TOR pathway and inhibits autophagy flux in THP-1 cells. As expected, NC treatment significantly decreased the expression of p62, p-p70S6K, p-mTOR and PI3K as compared with those in cells treated with ox-LDL alone. The level of LC3-II in NC-treated group was markedly higher than that in ox-LDL group. However, these effects of NC were eliminated by treatment with 740Y-P, an activator of PI3K. Taken together, these results indicate that NC abolishes ox-LDL-induced PI3K/m-TOR activation and consequently restores autophagy flux in ox-LDL-treated THP-1 cells.

## Discussion

Macrophage foam cell formation plays a critical role in the development of atherosclerosis. Our previous studies indicated that NC, a curcumin derivate, has beneficial effects in prevention of atherosclerosis [21]. However, the underlying mechanisms remain unclear. In the present study, we found that autophagy prevented foam cell formation in ox-LDL-treated THP-1 cells, and NC normalized the autophagy flux likely through a PI3K-Akt/mTOR-dependent mechanism. Furthermore, we conclude that NC reduces foam cell formation in ox-LDL-induced THP-1 cells through restoring autophagy flux.

Autophagy was found to play critical roles in lipid metabolism and the pathogenesis of atherosclerosis [29, 30]. In present study, we evaluated the influence of ox-LDL on autophagy flux by determining the LC3II and p62 levels in THP-1 cells. LC3, as a marker of autophagy, has two forms. LC3-I is mainly present in the cytoplasm and can be transformed into LC3-II when autophagy occurs. LC3-II is located on the membrane of AP and degraded by hydrolase in the lysosomes when AP is formed. P62, also known as SQSTM1, is a target and receptor for autophagy, which expression is usually assessed to evaluate the level of autophagy flux. In general, LC3II level positively correlates with AP numbers [31], but p62 level is negatively associated with autophagy flux [10, 11]. Our results revealed that ox-LDL significantly impaired autophagy flux in THP-1 cells with a decreased LC3II, and an elevated p62 level. The PI3K/Akt/mTOR signaling is an important pathway to regulate autophagy. It was observed that ox-LDL treatment markedly increased the levels of PI3K, p-mTOR, and p-p70S6K in THP-1 cells, which is in agreement with the previous study showing that ox-LDL inhibits autophagy via activating the PI3K/Akt/mTOR pathway in vascular smooth muscle cells. These data have shown that ox-LDL inhibits autophagy in THP-1 cells.

Next, we evaluated the effect of autophagy on foam cell formation and intracellular accumulation of total cholesterol in ox-LDL-treated THP-1 cells using autophagy inhibitor CQ and autophagy inducer rapamycin, respectively. Our results indicated that blocking autophagy flux increased Oil Red O-positive staining and intracellular total cholesterol contents in THP-1 cells in response to incubation with ox-LDL, while activation of autophagy exerted the opposite effect. These findings further confirmed that autophagy prevents intracellular lipid accumulation and foam cell formation [16, 32], suggesting that regulation of autophagy may serve as a novel and more effective approach to inhibiting and reversing atherosclerosis.

In the present study, we found that curcumin derivative NC restored autophagy flux and thus decreased foam cell formation in ox-LDL-treated THP-1 cells. Our western blotting results showed that 10 μM NC significantly increased the expression of LC3-II and lowered the level of p62 protein in ox-LDL-treated THP-1 cells, suggesting that NC can rescue impairment of autophagy flux induced by ox-LDL. Meanwhile, our TEM results also indicated that NC rescued autophagy in ox-LDL-treated THP-1 cells as indicated by an increase in APs and ALs. Furthermore, the tandem mCherry-GFP-LC3 reporter assay confirmed that NC obviously facilitated the process of autophagy flux in ox-LDL-treated THP-1 cells. Taken together, these data have revealed that NC rescues the impaired autophagy flux in ox-LDL-treated THP-1 cells.

Then we further explored the underlying mechanism of NC restoration of autophagy flux in ox-LDL-induced THP-1 cells. Autophagy is negatively regulated by the PI3K/Akt/mTOR pathway. It has been shown that class-I PI3K is essential for mTOR-downstream signaling [33]. Activation of PI3K leads to Akt phosphorylation, and phosphorylated Akt subsequently promotes phosphorylation and activation of mTOR. In this study, we have revealed that ox-LDL blocks autophagy flux likely through activation of mTOR signaling, and NC rescues the impaired autophagy flux in ox-LDL-treated THP-1 cells. Furthermore, other studies have indicated that curcumin induces autophagy via inhibiting the PI3K/Akt/mTOR pathway [34, 35]. However, it was previously unknown whether NC, a curcumin derivative, also facilitates autophagy flux through the same mechanism as curcumin. In the present study, NC significantly inhibited ox-LDL-induced PI3K/Akt/mTOR activation and impairment of autophagy flux in THP-1 cells, as indicated by the reduced levels of PI3K, p-mTOR, p-p70S6K and p62, and an increase in LC3-II level. Notably, the above effects of NC were abolished by treatment with 740Y-P, an activator of PI3K. These data suggest that NC restores autophagy flux in ox-LDL-treated THP-1 cells likely via inhibition of the PI3K/Akt/mTOR pathway.

Here, we have clearly demonstrated that NC inhibits foam cell formation in ox-LDL-treated THP-1 cells though restoring autophagy flux. Recently, several studies have indicated that autophagy facilitates intracellular lipid droplet degradation and cholesterol efflux and thus impedes foam cell formation [16, 17, 18]. In the current study, we first determined the effects of NC on foam cell formation and intracellular ox-LDL accumulation. Our results showed that NC greatly decreased intracellular lipid aggregation (Fig 3A and 3B) and foam cell formation in THP-1 cells (Fig 3C and 3F). It is well known that intracellular cholesterol ester content is associated with lipid uptake and cholesterol efflux. To further confirm that NC attenuates foam cell formation through rescuing autophagy flux, we examined the colocalization of LDs with autophagosomal marker LC3-II. We found that a decrease in LDs was almost identical no matter whether THP-1 cells were co-treated with NC and ox-LDL or consecutively treated with ox-LDL and then NC, suggesting that NC lowers ox-LDL accumulation in THP-1 cells not through decreasing ox-LDL uptake. Importantly, the results from the current study clearly demonstrated that NC significantly decreases the levels of total cholesterol and cholesterol esters, and increases the cholesterol efflux to apoA-I and HDL in THP-1 cells exposed to ox-LDL cholesterol. However, the above protective effects of NC against THP-1 foam cell formation could be abolished by 3-MA, a lysosome-independent inhibitor of autophagy, or CQ, an autophagy-lysosome inhibitor. These data indicate that NC prevents THP-1 foam cell formation through restoring autophagy flux that promotes the degradation of LDs to generate free cholesterol, which is mainly for ABCA-1 dependent efflux.

In conclusion, the results from the present study show that autophagy flux is impaired in ox-LDL-treated THP-1 cells, leading to a decrease in the degradation of ox-LDL and intracellular accumulation of lipids. Most importantly, we have demonstrated that NC rescues the impaired autophagy flux likely through inhibiting the PI3K/mTOR pathway, which may facilitate lipophagy and cholesterol efflux and ultimately attenuates the foam cell formation in ox-LDL-treated THP-1 cells. Therefore, our study provides a novel mechanism for NC to prevent THP-1 foam cell formation and highlights the regulation of autophagy as a promising therapeutic avenue for treatment of atherosclerosis.

## References

[1] Barascuk N, Skjot-Arkil H, Register TC, Larsen L, Byrjalsen I, Christiansen C, et al Human macrophage foam cells degrade atherosclerotic plaques through cathepsin K mediated processes. BMC cardiovascular disorders 2010; 10. 10.1186/1471-2261-10-19 PMC2868786 20409295

[2] Suzuki H, Kurihara Y, Takeya M, Kamada N, Kataoka M, Jishage K, et al A role for macrophage scavenger receptors in atherosclerosis and susceptibility to infection. Nature 1997; 386(6622). 10.1038/386292a0 9069289

[3] Doonan RJ, Hafiane A, Lai C, Veinot JP, Genest J, Daskalopoulou SS. Cholesterol efflux capacity, carotid atherosclerosis, and cerebrovascular symptomatology. Arteriosclerosis, thrombosis, and vascular biology 2014; 34(4). 10.1161/ATVBAHA.113.302590 24558111

[4] Duarte JH. Atherosclerosis: cholesterol efflux capacity-a new biomarker for cardiovascular risk? Nature reviews Cardiology 2015; 12(1). 10.1038/nrcardio.2014.198 25445136

[5] Gupta A. Cholesterol efflux capacity and atherosclerosis. The New England journal of medicine 2011; 364(15). 10.1056/NEJMc1101853 21488781

[6] Weibel GL, Drazul-Schrader D, Shivers DK, Wade AN, Rothblat GH, Reilly MP, et al Importance of evaluating cell cholesterol influx with efflux in determining the impact of human serum on cholesterol metabolism and atherosclerosis. Arteriosclerosis, thrombosis, and vascular biology 2014; 34(1). 10.1161/ATVBAHA.113.302437 PMC4005807 24202308

[7] Nguyen SD, Oorni K, Lee-Rueckert M, Pihlajamaa T, Metso J, Jauhiainen M, et al Spontaneous remodeling of HDL particles at acidic pH enhances their capacity to induce cholesterol efflux from human macrophage foam cells. Journal of lipid research 2012; 53(10). 10.1194/jlr.M028118 PMC3435544 22855736

[8] Fan W, Tang Z, Chen D, Moughon D, Ding X, Chen S, et al Keap1 facilitates p62-mediated ubiquitin aggregate clearance via autophagy. Autophagy 2010; 6(5). 10.4161/auto.6.5.12189 PMC4423623 20495340

[9] Mochida K, Oikawa Y, Kimura Y, Kirisako H, Hirano H, Ohsumi Y, et al Receptor-mediated selective autophagy degrades the endoplasmic reticulum and the nucleus. Nature 2015; 522(7556). 10.1038/nature14506 26040717

[10] Klionsky DJ, Abdalla FC, Abeliovich H, Abraham RT, Acevedo-Arozena A, Adeli K, et al Guidelines for the use and interpretation of assays for monitoring autophagy. Autophagy; 2012; 8(4). 10.4161/auto.19496 PMC3404883 22966490

[11] Sarkar S, Korolchuk V, Renna M, Winslow A, Rubinsztein DC. Methodological considerations for assessing autophagy modulators: a study with calcium phosphate precipitates. Autophagy 2009; 5(3). 10.4161/auto.5.3.7664 19182529

[12] Singh R, Kaushik S, Wang Y, Xiang Y, Novak I, Komatsu M, et al Autophagy regulates lipid metabolism. Nature 2009; 458(7242). 10.1038/nature07976 PMC2676208 19339967

[13] Zhang YL, Cao YJ, Zhang X, Liu HH, Tong T, Liu CF, et al The autophagy-lysosome pathway: a novel mechanism involved in the processing of oxidized LDL in human vascular endothelial cells. Biochemical and biophysical research communications 2010; 394(2). 10.1016/j.bbrc.2010.03.026 20223224

[14] Ouimet M, Franklin V, Mak E, Liao X, Tabas I, Marcel YL. Autophagy regulates cholesterol efflux from macrophage foam cells via lysosomal acid lipase. Cell metabolism 2011; 13(6). 10.1016/j.cmet.2011.03.023 PMC3257518 21641547

[15] Mei S, Gu H, Ward A, Yang X, Guo H, He K, et al p38 mitogen-activated protein kinase (MAPK) promotes cholesterol ester accumulation in macrophages through inhibition of macroautophagy. The Journal of biological chemistry 2012; 287(15). 10.1074/jbc.M111.333575 PMC3320924 22354961

[16] Li BH, Yin YW, Liu Y, Pi Y, Guo L, Cao XJ, et al TRPV1 activation impedes foam cell formation by inducing autophagy in oxLDL-treated vascular smooth muscle cells. Cell death & disease 2014; 5. 10.1038/cddis.2014.146 PMC4001301 24743737

[17] Kim HS, Montana V, Jang HJ, Parpura V, Kim JA. Epigallocatechin gallate (EGCG) stimulates autophagy in vascular endothelial cells: a potential role for reducing lipid accumulation. The Journal of biological chemistry, 2013; 288(31). 10.1074/jbc.M113.477505 PMC3829354 23754277

[18] Han J, Pan XY, Xu Y, Xiao Y, An Y, Tie L, et al Curcumin induces autophagy to protect vascular endothelial cell survival from oxidative stress damage. Autophagy 2012; 8(5). 10.4161/auto.19471 22622204

[19] Zhou GZ, Sun GC, Zhang SN. The Interplay between Autophagy and Apoptosis Induced by One Synthetic Curcumin Derivative Hydrazinobenzoylcurcumin in A549 Lung Cancer Cells. Journal of biochemical and molecular toxicology 2015; 29(6). 10.1002/jbt.21694 25683568

[20] Mourtas S, Lazar AN, Markoutsa E, Duyckaerts C, Antimisiaris SG. Multifunctional nanoliposomes with curcumin-lipid derivative and brain targeting functionality with potential applications for Alzheimer disease. European journal of medicinal chemistry 2014; 80. 10.1016/j.ejmech.2014.04.050 24780594

[21] Gong YZ, Yao HL, Sun SW, Song LP, Li RD, Liao DF, et al SREBP-1/Caveolin-1 Mediate the Anti-atherosclerotic Effect of Curcumin Nicotinate in Apolipoprotein E-Deficient Mice. Chinese Journal of Arteriosclerosis 2014; 22 (12).

[22] Zhang CP, Sun SW, Gong YZ, Ou L, Lin LM, Liao DF, et, al PCSK9/LDLR Pathway Mediates Curcumin Trinicotinate Promoting Lipid Uptake of HepG2. Progress in Biochemistry and Biophysics 2015; 42(9).

[23] Zhang M, Wu JF, Chen WJ, Tang SL, Mo ZC, Tang YY, et al MicroRNA-27a/b regulates cellular cholesterol efflux, influx and esterification/hydrolysis in THP-1 macrophages. Atherosclerosis 2014; 234 (1). 10.1016/j.atherosclerosis.2014.02.008 24608080

[24] de Bem AF, Farina M, Portella Rde L, Nogueira CW, Dinis TC, Laranjinha JA, et al Diphenyl diselenide, a simple glutathione peroxidase mimetic, inhibits human LDL oxidation in vitro. Atherosclerosis 2008; 201(1). 10.1016/j.atherosclerosis.2008.02.030 18440006

[25] Cheng LC, Su KH, Kou YR, Shyue SK, Ching LC, Yu YB, et al alpha-Lipoic acid ameliorates foam cell formation via liver X receptor alpha-dependent upregulation of ATP-binding cassette transporters A1 and G1. Free radical biology & medicine, 2011; 50(1). 10.1016/j.freeradbiomed.2010.10.706 21034810

[26] Linder MD, Mayranpaa MI, Peranen J, Pietila TE, Pietiainen VM, Uronen RL, et al Rab8 regulates ABCA1 cell surface expression and facilitates cholesterol efflux in primary human macrophages. Arteriosclerosis, thrombosis, and vascular biology 2009; 29(6). 10.1161/ATVBAHA.108.179481 19304576

[27] Tian GP, Chen WJ, He PP, Tang SL, Zhao GJ, Lv YC, et al MicroRNA-467b targets LPL gene in RAW 264.7 macrophages and attenuates lipid accumulation and proinflammatory cytokine secretion. Biochimie 2012; 94(12). 10.1016/j.biochi.2012.08.018 22963823

[28] Gu HF, Nie YX, Tong QZ, Tang YL, Zeng Y, Liao DF, et al Epigallocatechin-3-gallate attenuates impairment of learning and memory in chronic unpredictable mild stress-treated rats by restoring hippocampal autophagic flux. PLoS One 2014; 9(11). 10.1371/journal.pone.0112683 PMC4231069 25393306

[29] Liao X, Sluimer JC, Wang Y, Subramanian M, Brown K, Pattison JS, et al Macrophage autophagy plays a protective role in advanced atherosclerosis. Cell metabolism 2012; 15(4). 10.1016/j.cmet.2012.01.022 PMC3322248 22445600

[30] Maiuri MC, Grassia G, Platt AM, Carnuccio R, Ialenti A, Maffia P. Macrophage autophagy in atherosclerosis. Mediators of inflammation 2013. 10.1155/2013/584715 PMC3563164 23401644

[31] Wang AL, Boulton ME, Dunn WA Jr., Rao HV, Cai J, Lukas TJ, et al Using LC3 to monitor autophagy flux in the retinal pigment epithelium. Autophagy 2009; 5(8). 10.4161/auto.5.8.10087 PMC3704326 19855195

[32] Wang X, Li L, Niu X, Dang X, Li P, Qu L, et al mTOR enhances foam cell formation by suppressing the autophagy pathway. DNA and cell biology 2014; 33(4). 10.1089/dna.2013.2164 PMC3967384 24512183

[33] Zhao R, Chen M, Jiang Z, Zhao F, Xi B, Zhang X, et al Platycodin-D Induced Autophagy in Non-Small Cell Lung Cancer Cells via PI3K/Akt/mTOR and MAPK Signaling Pathways. Journal of Cancer 2015; 6(7). 10.7150/jca.11291 PMC4466411 26078792

[34] Zhao Z, Li C, Xi H, Gao Y, Xu D. Curcumin induces apoptosis in pancreatic cancer cells through the induction of forkhead box O1 and inhibition of the PI3K/Akt pathway. Molecular medicine reports 2015; 12(4). 10.3892/mmr.2015.4060 26166196

[35] Xu X, Qin J, Liu W. Curcumin inhibits the invasion of thyroid cancer cells via down-regulation of PI3K/Akt signaling pathway. Gene 2014; 546(2). 10.1016/j.gene.2014.06.006 24910117

